# A Process Evaluation of a Performance Psychology Intervention for Transitioning Elite and Elite Musicians

**DOI:** 10.3389/fpsyg.2020.01090

**Published:** 2020-05-26

**Authors:** Jolan Kegelaers, Raôul R. D. Oudejans

**Affiliations:** ^1^Faculty of Sports and Nutrition, Amsterdam University of Applied Sciences, Amsterdam, Netherlands; ^2^Faculty of Psychology and Educational Sciences, Vrije Universiteit Brussel, Brussels, Belgium; ^3^Department of Human Movement Sciences, Amsterdam Movement Sciences, Institute for Brain and Behaviour Amsterdam, Vrije Universiteit Amsterdam, Amsterdam, Netherlands

**Keywords:** deliberate practice, focus of attention, imagery, implementation research, performing under pressure, self-regulation

## Abstract

The present study presents a process evaluation of a performance psychology intervention for transitioning elite and elite musicians. The goal of the intervention was to provide participants with an amalgamation of evidence-informed principles, aimed to improve their quality of practice and performance preparation. The intervention consisted of an educational session followed by four workshops. In total, eight transitioning elite and seven elite musicians participated. Process measures included quantitative and qualitative workshop evaluations, monitoring logs, and semi-structured interviews. Overall, the intervention was evaluated positively by the participants. However, differences were present between the groups, with the elite musicians typically evaluating the intervention more favorably compared to the transitioning elites. Specific positive outcomes included an increased awareness and re-examining of current practice strategies, more structured and goal-directed practice, increased practice efficiency and focus, a more proactive approach to performances, and increased attention for the physical aspects of playing. Moreover, a number of contextual considerations and implementation challenges became evident. Important implications for performance psychology interventions and practitioners in music are discussed.

## Introduction

It is increasingly recognized that performance psychology can provide an important added value for classical musicians’ development and performance (Pecen et al., 2016; Sly et al., 2019). This is evidenced by a rise in studies looking at the implementation of performance psychology interventions – often derived from sport psychology – in a musical context. Many of these interventions have been directed toward reducing music performance anxiety. Performance anxiety is recognized as a debilitating performance factor (Kenny, 2011), which many classical musicians experience during all phases of their career (Papageorgi et al., 2013). Scholars have demonstrated the effectiveness of psychological interventions (e.g., relaxation techniques, self-talk, cognitive restructuring) in decreasing self-reported anxiety (Clark and Williamon, 2011; Hoffman and Hanrahan, 2012; Osborne et al., 2014; Braden et al., 2015; Spahn et al., 2016; Cohen and Bodner, 2019) as well as improving performance (Hoffman and Hanrahan, 2012; Spahn et al., 2016; Cohen and Bodner, 2019).

However, it has been argued that psychological interventions overly focus on decreasing performance anxiety and addressing its symptoms – which might not always be realistic nor desirable. As such, these interventions often fail to address how optimal focus of attention, functioning, and performance under pressure might be facilitated (Pecen et al., 2016; Oudejans et al., 2017; Cohen and Bodner, 2019). Furthermore, to develop expertise, musicians have to engage in thousands of hours of deliberate practice (i.e., effortful, focused, goal-directed) (Ericsson and Harwell, 2019). Such prolonged periods of practice are associated with a number of additional challenges, including practice efficiency (Duke et al., 2009), motivational constraints (Ericsson, 2008), decreased psychological well-being (Kenny et al., 2014; Kegelaers et al., in press), and overuse injuries (Bird, 2013; Baadjou et al., 2016). Many of these challenges might be aggravated or intensified by an enduring focus of music education on high volumes of practice (i.e., quantity) rather than on quality of practice (Hatfield, 2016; Pecen et al., 2016). In contrast, evidence suggests that quantity of practice in itself may sometimes be unrelated (Williamon and Valentine, 2000; Duke et al., 2009) or even inversely related to musical performance (Bonneville-Roussy and Bouffard, 2015). Performance psychology could, therefore, also provide insights into more effective practice and performance preparation strategies for musicians (Clark and Williamon, 2011; Pecen et al., 2016).

A limited number of studies have looked at how psychological interventions can improve musicians’ practice activities. For example, Clark and Williamon (2011) evaluated a mental skills training (MST) program centered around motivation and effective practice (e.g., goal-setting, time management), relaxation and arousal control (e.g., self-talk, relaxation), and performance preparation and enhancement (e.g., imagery, focus of attention). The authors found that following the intervention, participants scored higher on self-regulation and reported increased self-awareness of performance preparation, increased practice efficiency, shifts in views toward anxiety, and more positive attitudes toward music making (Clark and Williamon, 2011). More recently, Hatfield (2016) examined the effectiveness of a MST program on music students’ practice behaviors. Results of this preliminary intervention highlighted that the participants developed important self-regulating skills (e.g., goal setting, imagery, arousal-regulation, concentration, self-observation). In turn, participants believed that these skills helped them to be more deliberate, structured, focused, and goal-directed in their practice; as well as more confident and non-judgmental in their performances (Hatfield, 2016).

As performance psychology in music is growing, several avenues for further research become visible. First, most interventions have been set up as straightforward pre- and post-test evaluations; with (e.g., Hoffman and Hanrahan, 2012; Spahn et al., 2016) or without (e.g., Osborne et al., 2014; Bakker et al., 2016) a control group. Although such designs are useful to demonstrate intervention effects, they do not provide much information into how these interventions are received, how they are delivered most effectively, or how they can be improved (Clark and Williamon, 2011). Pecen et al. (2016) already pointed out that certain challenges exist – informed by culture or tradition – when delivering performance psychology principles within music. Indeed, the limited intervention studies that included some form of qualitative evaluation (Clark and Williamon, 2011; Hatfield, 2016) reported both the ups and downs of the intervention application as well as potential avenues for future improvement. Advancing this research further, there is a need for process evaluations of performance psychology interventions in music (Moore et al., 2015; Randall et al., 2019). Such process evaluations are research strategies aimed to “gather information about actions taken and stakeholder perceptions (from those targeted by the intervention and from those involved in its design and delivery) of the quality of intervention components and activities” (Randall et al., 2019, p. 50).

Second, existing performance psychology interventions have primarily focused on music students, with limited attention for musicians situated in other career phases (e.g., transitioning elite or elite musicians; Pecen et al., 2018). Such interventions for students are typically delivered within a formal education setting. Less is known about how interventions can be set up and are received within less formal educational or professional settings. Moreover, different career phases come with their own distinct challenges and barriers. For example, the transition from music education into the professional field is typically anchored by highly stressful auditions (Kenny et al., 2014). Furthermore, this transition is often also marked by a steep increase in deliberate practice (MacNamara et al., 2006). As transitioning elite musicians eventually enter the professional field, they might face excessively high workloads and financial pressure; often combined with a new need to balance their profession with family life, reducing the time available for practice (MacNamara et al., 2006). Finally, as musicians grow older, they might perceive increasing difficulties sustaining their own level of performance (MacNamara et al., 2006; MacRitchie and Garrido, 2019). Given these changing demands, transitioning elite and elite musicians might equally benefit from performance psychology interventions.

The purpose of the present exploratory mixed methods study was, thus, to conduct a process evaluation of a multimodal evidence-informed performance psychology intervention. The aim of this intervention was to use performance psychology principles to improve musicians’ overall quality of practice and performance preparation. As such, it advances existing intervention studies focusing primarily on teaching strategies to cope with performance anxiety. The current study is based on an existing intervention, which has been successfully delivered with conservatory music students (Bakker et al., 2016). This earlier intervention was adapted for and delivered to transitioning elite and elite musicians. The principles included within the intervention are outlined below.

## Intervention Background

The intervention consisted of an educational session – providing the scientific background for the intervention – followed by four workshops. The aim of these workshops was to provide participants with an amalgamation of evidence-informed principles they can selectively use in their personal practice or performance preparation, based on personal preferences. The principles included within this intervention were selected based on recent advances in music psychology research, as well as a number of other performance domains including sports, work, and law enforcement.

Workshop 1 focused on the use of *deliberate practice*. The idea of deliberate practice was first introduced by Ericsson et al. (1993), who stressed the importance of quality of practice to develop expertise. The authors described deliberate practice as highly structured and goal-directed practice activities, executed with maximum concentration and effort, with the explicit aim to improve performance. Recent evidence highlights the importance of musicians’ ability to structure and manage practice time and self-regulate effectively in order to engage in quality deliberate practice (e.g., Bonneville-Roussy and Bouffard, 2015; Hatfield et al., 2017). With regards to time management, Ericsson et al. (1993) argued that engagement in high-quality deliberate practice might only be sustainable for a limited amount of time. Contrary to traditional practice in music (Pecen et al., 2016), some music educators (e.g., Klickstein, 2009) have, therefore, proposed practice routines consisting of consecutive relatively short practice blocks (e.g., 20 min), followed by short breaks. Previous research found that such time management strategies were perceived as highly valuable and effective by music students (Bakker et al., 2016). Furthermore, frequent breaks can also help prevent overuse injuries in musicians (Baadjou et al., 2016).

To engage in high quality individual deliberate practice, musicians also need to be self-regulating in their practice. Based on the work of Zimmerman (2000), self-regulation refers to an individual’s active metacognitive engagement in one’s own learning process. It is typically presented as a cyclical multiphase process; consisting of a forethought phase (i.e., goal-setting and planning), a performance phase (i.e., self-control and self-monitoring), and reflection phase (i.e., reflection on progress). Self-regulation allows musicians to select, monitor, and adjust their practice strategies (Concina, 2019; McPherson et al., 2019). Research has consistently demonstrated that effective learners use such self-regulating strategies more frequently, both in music (e.g., Bonneville-Roussy and Bouffard, 2015; Hatfield et al., 2017) and other performance domains (e.g., Cleary and Zimmerman, 2001).

Workshop 2 centered on the use of *imagery*. Musical imagery is defined as “a multimodal process by which an individual generates the mental experience of auditory features of musical sounds, and/or visual, proprioceptive, kinesthetic, and tactile properties of music related movements” (Keller, 2012, p. 206). Several psychological intervention studies already effectively used imagery as a way to prepare for performances and decrease performance anxiety (e.g., Hoffman and Hanrahan, 2012; Hatfield, 2016). However, imagery can also serve to promote other aspects of music performance (e.g., temporal efficiency, movement economy, expressivity) (Keller, 2012; DeSantis et al., 2019). The present workshop focused on teaching musical imagery through different modalities (e.g., auditory, visual, kinesthetic) and in relation to different functions (e.g., technical execution, expressiveness, performance).

Workshop 3 revolved around *focus of attention*; or more specifically the promotion of an external focus of attention. Research in sports (e.g., Bell and Hardy, 2009) has demonstrated that the use of an external focus of attention (i.e., on intended movement effect) – compared to an internal focus of attention (i.e., on body movement itself) – has positive effects on the learning and execution of complex motor skills (Wulf and Lewthwaite, 2016). In music, evidence exists that adopting an external focus (i.e., imagining expression, emotion, images, etc.) – compared to an internal focus (i.e., focus on technical execution, fingerings, etc.) – increases movement effectiveness and learning efficiency (Williams, 2019). Furthermore, studies using expert ratings of music performance, found that skilled musicians adopting an external focus of attention are generally rated higher on expressiveness and perceived musical ability (Van Zijl and Luck, 2013; Mornell and Wulf, 2019). Finally, research has demonstrated that increased pressure (e.g., during important performances) can lead to a shift in attentional focus toward irrelevant stimuli (e.g., worrying thoughts; Buma et al., 2015; Oudejans et al., 2017). As such, adopting an external focus can also enhance performance in pressure situations, by directing attention to more task-relevant stimuli (Bell and Hardy, 2009; Williams, 2019).

Workshop 4 focused on *performance preparation*. Within this workshop, two principles were addressed. The first principle concerned scenario planning, involving the anticipation and preparation for potential negative scenarios or setbacks. Research within sports (e.g., Kegelaers and Wylleman, 2019) and work (e.g., Chermack, 2005) has demonstrated that scenario planning can increase resilience against pressure, by strengthening an individuals’ personalized coping strategies to effectively prevent and/or manage stressful or unexpected conditions. The second principle related to training under pressure. Kenny et al. (2014) found that many elite musicians used performance simulation (e.g., playing for other people) as a form of performance preparation. Wan and Huon (2005) demonstrated that such practicing under pressure can improve musical performance under ‘real-life’ pressure conditions. Similar results have also been found for pressure training through the use of augmented reality in music (Williamon et al., 2014) and are consistent with findings from other performance domains (e.g., Oudejans and Pijpers, 2010; Nieuwenhuys and Oudejans, 2011). This workshop used the planned disruptions framework (Kegelaers et al., 2020) to explore how musicians can create pressure situations within their own practice, considering manipulation of demands in relation to the individual, the environment, or the task at hand.

## Materials And Methods

### Design

Process evaluations typically adopt a combination of different quantitative and qualitative research methods (Moore et al., 2015). As such, this approach fits firmly within a pragmatic research paradigm (Giacobbi et al., 2005), characterized by ontological relativism (Poucher et al., 2019). In line with our pragmatic research paradigm and process evaluation approach, an explanatory mixed methods design was used for the present study, whereby qualitative data was used to build on, interpret, and explain the quantitative data (Creswell and Plano Clark, 2017).

### Participants

Participants in the present study included both transitioning elite and elite musicians. Transitioning elite musicians (*n* = 8) included fellows of the academy of a world-renowned international orchestra (7 female; 1 male), with a mean age of 23.8 (*SD* = 1.4) and an average of 18.1 years of experience as a musicians (*SD* = 2.0). This academy allowed the musicians to work embedded within the orchestra, among other learning opportunities, for the duration of 1 year; with the specific aim to prepare them for the transition into the professional field. During this year, the transitioning elite musicians also engage in a mentorship program with established orchestra members. Instruments played by the transitioning elites included violin (*n* = 3), cello, viola, clarinet, bass, and harp (all *n* = 1). Elite musicians (*n* = 7) included members of one accomplished professional international orchestra (4 female; 3 male). They had a mean age of 44.3 (*SD* = 13.7), with 35.4 years of experience (*SD* = 11.3). Instruments played by the elites included violin (*n* = 2), cello (*n* = 2), viola, clarinet, and bass (all *n* = 1). All participants had previously obtained bachelor’s or master’s degrees in music education.

### Procedure

Prior to the start of this project, ethical approval was obtained through the authors’ institutional ethics committee. Contact with the individual participants was first established through the orchestras involved in this project. For the transitioning elite musicians, the intervention was offered as part of the curriculum of the academy, whereas elite musicians all actively enrolled in the intervention. As highlighted in *Intervention Background* section, a theoretical introduction session was followed by four workshops. The workshops are summarized in Table 1. All sessions and workshops were organized separately for both groups. Informed consents were collected from all participants during the introduction session. Due to practical reasons, Workshops 1 and 2 and Workshops 3 and 4 were always delivered on the same day. The workshops were delivered by both authors collaboratively, who have a background in sport and performance psychology. Mentors of the transitioning elite musicians were also invited to attend the workshops to facilitate the transfer of the intervention principles to the day-to-day work of the participants. In total, around two to four mentors attended each workshop. In line with the recommendations of Clark and Williamon (2011), scientific background information was kept to a minimum, and participant interaction and discussion was actively encouraged during the workshops. Factsheets were also provided at the end of each workshop, providing both the theoretical background of each workshop, as well as some practical exercises which participants were asked to try out during their daily practice. Around 1 week after each workshop, participants were asked to complete a preliminary workshop evaluation.

**TABLE 1:** Outline of the intervention components.

Workshop	Workshop components	Key references
Deliberate practice	Time management	Ericsson et al., 1993; Klickstein, 2009; Bakker et al., 2016
	Self-regulation	Zimmerman, 2000; Hatfield et al., 2017; McPherson et al., 2019
Imagery	Imagery	Keller, 2012; DeSantis et al., 2019
Focus of attention	External focus	Wulf and Lewthwaite, 2016; Mornell and Wulf, 2019; Williams, 2019
Performance preparation	Scenario planning	Chermack, 2005; Kegelaers and Wylleman, 2019
	Planned disruptions	Wan and Huon, 2005; Nieuwenhuys and Oudejans, 2011; Kegelaers et al., in press

Following the workshops, weekly individual monitoring sessions were organized with the research assistants. These sessions lasted 30 min on average and had the dual function of providing individualized follow-up after the workshops, as well as providing a data collection moment to track participants’ progress. Logs were kept by the research assistants for all session. The first sessions were always organized in person; whereas the subsequent sessions could be either done in person or through telephone or Skype, dependent on the availability of the participants. The monitoring sessions were organized for 10 weeks. However, due to practical reasons (e.g., prolonged stay abroad) it was not possible to organize weekly monitoring sessions with all participants. In these cases, participants were provided the opportunity to deliver any questions or remarks via mail. In total, 66 sessions were organized with the 8 transitioning elite musicians and 46 sessions with the 7 elite musicians. The end of the monitoring sessions was anchored by performance moments. For the transitioning elite group, this performance moment entailed a mock audition organized by the academy. For the elite group, the end of the intervention was marked by the final week of performances during the orchestra season.

After the end of the intervention, all participants were invited for a concluding interview. These interviews were scheduled in a quiet place of the participants’ choice and led by the first author, who has extensive experience in qualitative research. Due to practical limitations, two interviews (1 transitioning elite, 1 elite) were done through Skype. Prior to the interviews, participants were also asked to complete the general intervention evaluation. At the conclusion of the interview, participants were thanked for their participation and asked how they would like to be informed about the further results of the project.

### Material

#### Quantitative Measures

1. *Quantitative preliminary workshop evaluations* were gathered with all participants, roughly one week after each workshop. These evaluations always included statements related to the usefulness of the workshop as a whole, each of the principles included in the workshop, the practical exercises, and the factsheets. Answers were rated on a 5-point Likert scale, ranging from 1 (“Totally disagree”) to 5 (“Totally agree”).
2. A *general intervention evaluation* was included at the end of the intervention. This evaluation included both general questions related to the whole intervention, as well as specific questions for each workshop component included in the intervention (see Table 1). The general questions included the statements: “I believe the project as a whole was a valuable experience,” “The workshops were valuable,” “I learned a lot from this project,” “I will continue to use the methods presented in the project,” “the monitoring sessions were useful,” and “the factsheets were useful.” Specific questions for each workshop component included: “I used this method frequently” and “I found this method valuable to reach my goals.” All answers were rated on a 5-point Likert scale, ranging from 1 (“Totally disagree”) to 5 (“Totally agree”).

#### Qualitative Measures

1. *Qualitative preliminary workshop evaluations* were gathered with all participants, roughly 1 week after each workshop. In addition to the quantitative evaluations (see section Quantitative Measures), participants were presented two open-ended questions; “Where there specific things you liked during this workshop?” and “How might we improve this workshop in the future?”
2. *Monitoring logs* were kept by research assistants during all the monitoring sessions. These monitoring logs provided an indication of how frequently participants engaged with the different principles, their experiences and perceived challenges working with the principles, and descriptions of the individualized follow-up.
3. Individual *semi-structured interviews* were conducted with all participants following conclusion of the intervention. An interview guide was developed to give direction to the interviews. This interview guide included four parts; first participants were asked about their general experiences during the intervention (e.g., “Looking back, what was your general experience during the past project?”). Second, participants were asked specifically about their experiences with each of the intervention principles (e.g., “How was your experience working with goal-setting?”). Third, participants were asked about some of the experienced challenges during the intervention (e.g., “What were some of the challenges you experienced implementing the principles, if any?”). Finally, participants were asked about potential areas of improvement for the intervention (e.g., “How might we improve the project in the future?).

### Data Analysis

#### Quantitative Data Analysis

An exploratory quantitative approach was adopted in the present study to find out if the two expertise groups evaluated the intervention differently. Descriptive statistics were first calculated for all quantitative variables. Between group differences for the general intervention evaluation were assessed using independent sample *t*-tests. Additionally, independent sample *t*-tests were also used to assess differences between the TE and E group for the frequency of use and perceived value of the different workshop components.

#### Qualitative Data Analysis

The qualitative data was analyzed using inductive thematic analysis. This approach was chosen as it is an accessible method to discern, analyze, and report meaning patterns, whilst maintaining theoretical and analytical flexibility (Braun et al., 2016). The analysis was conducted following the guidelines outlined by Braun and Clarke (2006). First, written transcripts of the qualitative preliminary workshop evaluations, the monitoring logs and the interviews were carefully read and reread in order to familiarize with the data. Second, small segments of data were given a code representing its meaning. Third, these codes were inductively clustered into lower order themes and further refined into higher order themes. Finally, each theme was carefully provided with a label, which succinctly represents its broader scope. The analysis was conducted by the first author who had extensive experience in qualitative research. Throughout the analysis process, the second author acted as a critical friend (cf. Smith and McGannon, 2018). As a critical friend, he served to scrutinize the analysis made by the lead researcher and offer potential alternative interpretations, to ensure rigor and trustworthiness of the analysis (Smith and McGannon, 2018).

## Results

In line with our explanatory mixed methods research design (cf. Creswell and Plano Clark, 2017), quantitative results will be provided first. Subsequently, qualitative data will be presented to explain and elaborate on the quantitative results. Throughout the results section, transitioning elite and elite participants are identified using TE and E respectively.

### Quantitative Results

**FIGURE 1:**
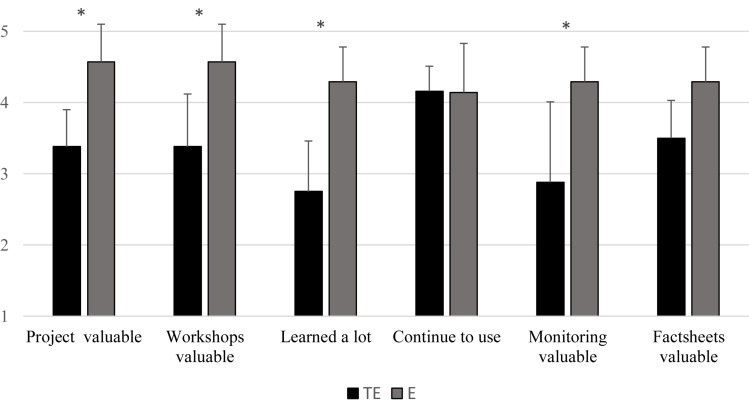
Mean values for the Overall intervention evaluations of the Transitioning elite (TE) and Elite (E) musicians ^∗^*p* < 0.05.

As indicated in the methods section, quantitative results were collected during preliminary workshop evaluations as well as the general intervention evaluation. However, due to space limitations, we only present results from the general intervention evaluation, especially as preliminary evaluations were consistent with the final evaluations. Evaluations of the intervention overall are summarized in Figure 1. The mean scores on the overall value of the project (on a 5-point scale) were rated significantly more favorably for the E group (*M* = 4.57, *SD* = 0.53) compared to the TE group (*M* = 3.38, *SD* = 0.52); *t*(13) = 4.40, *p* = 0.001, *d* = 2.26. Further independent sample *t*-tests also revealed significantly higher evaluation scores for the E group compared to the TE group for value of the workshops, *t*(13) = 3.53, *p* = 0.004, *d* = 1.82; whether they learned a lot, *t*(13) = 4.82, *p* < 0.001, *d* = 2.49; and value of the monitoring sessions, *t*(9.80) = 3.22, *p* = 0.009, *d* = 1.57.

**FIGURE 2:**
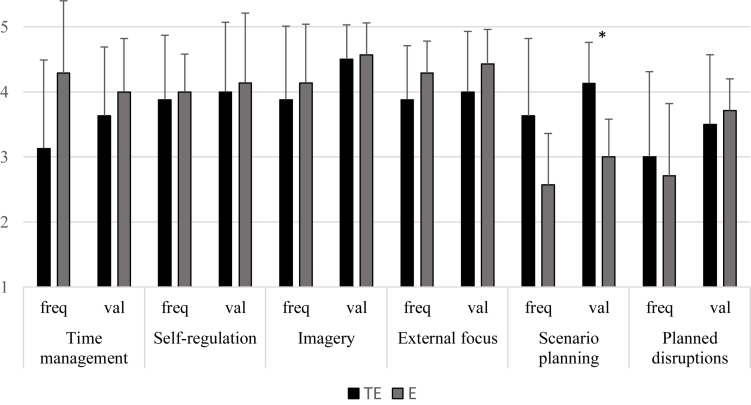
Mean values for Frequency of use and Perceived value of the different workshop components for the Transitioning elite (TE) and Elite (E) musicians. ^∗^*p* < 0.05.

Evaluations of the different workshop components in relation to frequency of use and perceived value are summarized in Figure 2. The frequency of use of the different components ranged between 3.00 and 3.88 for the TE group; and 2.57 and 4.29 for the E group. Independent sample *t*-tests found no significant differences in the frequency of use between the TE and the E groups (*p*s > 0.05). Although a substantial difference was found for the frequency of use of time management in the TE group (*M* = 3.13, *SD* = 1.36) compared to the E group (*M* = 4.29, *SD* = 1.11), this difference failed to reach significance; *t*(13) = −1.80, *p* = 0.096, *d* = 0.93. Similarly, a substantial difference was present in the use of scenario planning in the TE group (*M* = 3.63, *SD* = 1.19) compared to the E group (*M* = 2.57, *SD* = 0.79), which nevertheless failed to reach significance; *t*(13) = 1.99, *p* = 0.068, *d* = 1.04. Despite demonstrating only trends toward significance, both effect sizes did exceed the convention for a large effect (*d* = 0.80). The ratings for Perceived value of the workshop components ranged between 3.50 and 4.50 for the TE group; and 3.00 and 4.57 for the E group. A large significant difference was present in the perceived value of scenario planning between the TE group (*M* = 4.13, *SD* = 0.63) and the E group (*M* = 3.00, *SD* = 0.58); *t*(13) = 3.55, *p* = 0.004, *d* = 1.84. No additional significant differences were found for the perceived value of the different components between the TE and the E groups (*p*s > 0.05).

### Qualitative Results

The qualitative data indicated that participants evaluated the intervention as generally positive. In line with the quantitative findings, E musicians seemed to appraise the intervention somewhat more favorable compared to the TE group. Furthermore, participants also clearly differed in the way they adopted and evaluated the different sub-components of the intervention. This finding was consistent with the goal of the intervention, which was to present the participants with an amalgamation of principles they could selectively use based on their personal preferences. More detailed thematic analysis of the data resulted in three overarching themes: (a) *intervention outcomes*, (b) *positive intervention mechanisms*, and (c) *implementation challenges and improvements*. These findings are summarized in Table 2. Quotes are given throughout the results section marked by the participant’s number followed by “*I*,” “*M*,” or “*P*”; indicating that they were derived from the *interviews*, *monitoring logs*, or *preliminary qualitative evaluations* respectively.

**TABLE 2:** Summary of the qualitative results.

Intervention outcomes	Positive intervention mechanisms	Implementation challenges and improvements
Re-examine practice habits	Group interactions	Limited novelty (*TE*)
Increased awareness	Scientific yet practical approach	Time constraints (*TE*)
Goal-directed and structured	Monitoring sessions (*E*)	Mentor involvement (*TE*)
Increased practice efficiency and focus		Researchers’ background (*TE*)
Proactive approach to performances (*TE*)		Music as art
Attention for physical aspects of playing		Lack of stressful performances (*E*)

*(TE) or (E) indicates that this sub-theme was mentioned almost exclusively by this participant group.*

#### Intervention Outcomes

In total, six sub-themes were identified indicating the perceived positive outcomes gained from the intervention. The first broad theme was that it led participants to ***Re-examine practice habits*,** as highlighted by one E musician: “Yes, [the intervention] brought me a lot. It really inspired me to look differently at the whole aspect of studying and playing music” (E2-*I*). For several participants this re-examination helped to refresh some old or vague practice methods and make them more concrete: “I think a lot of those things weren’t necessarily new, but it was more like refreshing old methods and start thinking about those again. You’re going to do things again, because you haven’t done them in a long time.” (E6-*I*); “There were some things that I knew unconsciously, but of which I had never thought about very consciously. So, it was good to refresh that again and to think about what works for me” (TE7-*I*). In addition, some E musicians stated that re-examining their habits reinvigorated their motivation to practice as well: “It is really motivating because it gives you to feeling that you can still progress.” (E2-*I*); or “Normally, you get stuck into a pattern. Sometimes it is difficult to motivate yourself and you just have those bad days. So that way I also enjoyed working on it in a fresh way” (E6-*I*).

Related to re-examining practice habits, a second theme reflected an ***Increased awareness***, as illustrated by one musician: “Increased awareness, in general, I think was the most beneficial aspect of the intervention” (TE2-*I*). Among other things, participants stated that during the intervention they gained awareness of the types of goals they were setting in their practice: “It made me more, how to say, more aware. Aware of what goals I set myself, whether they are realistic and if maybe they put too much pressure on myself, or not enough” (TE4-*I*); “I’m more aware of how I’m practicing, what I’m doing and what I’m doing wrong. What are my goals? Because thanks to that I started to be aware of what I wanted to do more and more” (E6-*I*). As evidenced by this last quote, participants also became more aware of *what* they were doing and focusing on during practice. For example, some participants became more aware of the focus they were adopting during practice: “I was more aware when I was practicing internally [focussed]” (TE6-*M*). Finally, some participants also became more aware of when they were practicing inefficiently or unfocused: “It helped to notice thoughtlessness and automaticity” (TE1-*M*).

As a third theme, participants argued that they became more ***Structured and goal-directed*** in their practice. For example, several participants argued that they became much more structured in planning their practice material and organizing their practice time: “I just try to be very conscious about what comes next, not only those concerts in the next few weeks, but also to look further … I don’t know if I would have started this early before this intervention.” (E6-*I*). In addition to structuring their practice, some musicians also became more deliberate and goal-directed in their day-to-day practice activities. Participants argued that using specific practice goals, particularly helped to them in managing the different pieces they have to learn simultaneously: “It [the intervention] really helped to focus on smaller goals that make you study. It makes it more manageable.” (TE1-*M*); or

The goal-setting, for example, for me was really helpful for my organization. Because I have a lot of work and a lot of pieces to play. And if I know I have only about two or three hours before I have my next rehearsals. Sometimes that’s overwhelming and it puts a lot of pressure on me. And if you [plan] it before, it is really helping to structure it all and of course after you made a plan you normally feel less stressed. (TE4-*I*)

Finally, some participants also argued that reflection on their goals helped to inform future practice goals and activities: “I record much more of myself and I can reflect much more on what I have actually done. This also helps to set new goals. Thanks to this intervention, I really learned to do that” (E2-*I*).

A fourth perceived benefit of the intervention related to ***Increased practice efficiency and focus***. Several participants stated that they became more efficient in the way they practised: “I still have the feeling that I’m studying better now” (E6-*I*). More specifically, for most participants the intervention helped in maintaining an optimal focus during practice. This was, for example, referenced in relation to the deliberate practice strategies: “I particularly found that the structured practice, dividing the practice time into shorter, more manageable blocks, was very effective. Also planning exactly what I wanted to work on helped me to focus on what I needed to do most.” (E4-*I*); “I used the [structured practice] when I was tired and it worked quite well, because it contains very short practice blocks in which I could focus clearly.” (TE1-*I*). Furthermore, some participants also stated that the use of imagery improved focus: “For me [imagery] especially helped for my concentration. To make your body gets quiet, but your brain still can work how it wants to. It really helped me to focus.” (TE4-*I*). Finally, the use of external focus of attention was also referenced as a method to practice and play more effectively:

I think external focus is very important. I noticed while teaching and also while practicing myself, as soon as I think of something as an image or a long line, then it is so often that technical things solve themselves. This remains some kind of miracle for me. If you’re zooming in on the technical parts it won’t succeed, but when you think of it as a landscape it works out. Your body knows what to do and you just block it with thinking of technical elements. (E3-*I*)

The fifth theme concerned a ***Proactive approach to performances***. Especially the TE musicians reported that the intervention helped them to be more conscious and deliberate in preparing for stressful performances. This was often the result of using a combination of imagery, scenario planning, and planned disruptions. These strategies helped participants to feel more prepared for important performances and anticipate potential issues:

In combination with imagery or planned disruptions, I thought about the scenarios and actively searched for solutions. I then tried out how it would feel [to experience] those what-if scenarios. It helped me to feel the stress and feel that I can play under these circumstances. (TE8-*M*);

I used [imagery] a lot and certainly toward the audition, which also has to do with training under pressure and the what-if scenarios. Doing it every day a few times to prepare for everything. That helped for sure, because when I had my audition, I had already walked into the building a hundred times and all sorts of things went wrong. So that was really nice for practicing. (TE1-*I*)

In addition to feeling prepared, several participants also stated that the strategies helped them to be less stressed for performances and allowed them to adopt a more desirable (external) focus of attention: “I’m calmer in my head and can focus more on the interpretation of the music instead of difficult notes for example.” (TE2-*M*); “It helped to hide my nerves more. I felt more relaxed and my thoughts weren’t about internal focus anymore.” (TE4-*M*).

As a final theme, some participants – although mentioned least frequently – felt that the intervention helped in having more ***Attention for physical aspects of playing***. More specifically, several participants noted that the structured practice helped them in recognizing when they started feeling physical aches and forced them to take a break: “Sometimes I get problems with my arm and then it really helps that I practice for half an hour. Just to rest for a moment and to let go of the tension.” (E2-*M*); “I had some pain in my back, so the breaks were a good way to relax a bit while practicing.” (TE8-*M*). Furthermore, one participant also stated that controlling one’s focus of attention helped in being attentive to bodily sensations: “I really focused on something in my body and not just different focus in music. That helps a lot because then I’m a bit less tense in my body.” (TE5-*I*). Finally, the use of imagery was mentioned as a useful way to practice without physical strain: “I think it is also important because you can prevent yourself from being too physically involved.” (E3-*M*).

#### Positive Intervention Mechanisms

In addition to their general experiences, participants also mentioned several specific aspects they found beneficial during the intervention. First, most participants appreciated the way ***Group interactions*** were stimulated during the intervention: “I really liked that you brought people together and you talked about mental aspects and fears everyone has. It also helps to see that people have shared issues” (TE4-*P*). This group sharing might have helped in exchanging personal experiences, as well as alleviating some of the taboo on sharing information about individual practice time, which some participants believed to exist:

I thought that by interacting with each other you are keeping it more alive. So for that reason I think it has a lot of added value, that everybody is not doing it independently of each other, but that you are doing it with your colleagues at the same time. That way you can also talk about it. Because otherwise there also rests a little bit of a taboo on what you are doing at home. That is something you normally don’t talk about. (E3-*I*)

Additionally, most participants also seemed to appreciate the ***Scientific yet practical approach*** adopted in setting up the intervention. This was illustrated in the following quote:

It was nice because there was this structure in kind of scientific approach to it, which is missing a lot in music education in general. So, that was nice, because then you had some guidance and tools on how to do it and what to do and what not to do. That was nice. (TE3-*I*)

Participants also appreciated how these scientific principles were translated into practical exercises during the workshops: “I liked the fact that the things we were talking about, that we were right away experiencing them and trying these things out. And to share the point of view with the other academists and the mentors.” (TE8-*E*). Although the practical nature of the workshops was appreciated, several participants also argued even more exercises could have been included.

Thirdly, participants highlighted the importance of the ***Monitoring sessions*,** as it motivated them to continue using the techniques provided during the workshops: “I found it very nice that we would see each other every week. That was a motivation for me to study” (E2);

I really found the telephone calls very nice. I found them very inspiring and stimulating. It is actually very nice when someone is looking over your shoulder. Not like you had [four] workshops and then it is just “off you go.” (E5-*I*)

In addition to motivate participants to use the proposed principles, participants also appreciated the opportunity to ask questions and discuss challenges in applying the intervention principles, during the monitoring sessions: “I thought it was very nice that I could ask things and that we could discuss a number of things and search for solutions.” (TE1-*I*). These sentiments were shared by all E musicians. However, in line with the quantitative evaluations, the TE group seemed to be somewhat more critical of the monitoring sessions. Although most still perceived these sessions to be positive, the TE group also considered them to be an additional time constraint.

#### Implementation Challenges and Improvements

A number of challenges as well as potential avenues for improvement in the way the intervention was implemented, were mentioned by the participants. First, some participants argued that the intervention provided ***Limited novelty***. As mentioned before, several of the E musicians found the intervention useful to refresh or re-examine certain practice strategies. However, some TE musicians felt they had previously learned several of the principles: “It was actually not something really new. I met people who spoke about those things and mental train us and so it was not really new, I yeah, but I mean nevertheless it’s useful” (TE4-*I*). This might partially explain why the intervention as a whole was perceived less valuable by the TE musicians. Consequently, some participants argued that the included strategies should also be taught to music students: “I really think it’s perfect to give things like these [workshops] for conservatory students, because the things I already did, I learned partially from teachers” (TE7-*I*). Indeed, two E musicians stated that they began using these principles with their own students.

A second theme reflecting intervention challenges were the ***Time constraints*** experienced by the participants. Although the intervention principles were designed to make practice more efficient, participants argued that learning and incorporating them might actually require additional time: “I have the idea that it [Goal-setting] consumes more time. I know that it can save time, but it still feels like an extra addition while studying.” (E3-*I*). Especially the TE musicians frequently mentioned that due to their busy schedules, they lacked the time to try out these principles: “I had to prepare a two hours programme over the weekend, so I had no time to change my study routine.” (TE4-*M*);

Sometimes it felt that I preferred my own methods and not to try experimenting so much. But this kind of study will probably be very good for somebody who is in an Easter break of Christmas break or something where he can experiment I think. (TE3-*I*)

As highlighted by the last quote, the TE musicians, therefore, proposed that future interventions should carefully consider when such strategies might be best presented.

Thirdly, some TE participants also mentioned ***Mentor involvement*** as a potential intervention improvement. As stated, these established orchestra members were invited to attend and participate in the workshops as well. The TE participants largely appreciated this involvement, but at the same time argued that their involvement might have been larger and more structural:

If you can find a way to involve them [mentors] more. I think it was really nice that they were there actually, I just really liked it. Because you feel like, of course it’s a workshop and we are learning, but the fact that they are there too, we don’t feel like a student. We have a different level of course, they are professionals that are still learning, but it is nice that there’s not only students. (TE8-*I*);

Maybe it helps to work also closer together with the mentors. [During the workshops] we shared a lot of experiences and that really helped me also. Because of course they played for so long and had a lot of beautiful experiences which really helps. (TE4-*I*)

A fourth theme reflecting intervention challenges were the participants’ views on the ***Researchers’ background***. Both authors had a background primarily within sport and performance psychology rather than music education or music psychology. Some TE participants argued that the researchers’ lack of music background could hinder or undermine the transfer of knowledge during the workshops: “At a certain point you notice that it is very difficult to understand if you don’t do it yourself, because it [music] isn’t really a black and white matter.” (TE1-*I*). Consequently, the TE group suggested involving performance psychologists with a specific background in music: “It would have been nice if a musician would be on your team. This person could have explained it more in musical terms or slightly different to make it more understandable” (TE2-*I*). Notably, E participants did not consider this different background a hindrance and some even found it enriching: “It didn’t bother me that you had a background in sports. It didn’t make it less clear to us or made us question why these methods applied to us.” (E3-*I*); “I actually quite liked it that the [background] was different, but that it was still possible to have a conversation and an exchange of advice” (E5-*I*).

Fifth, another perceived challenge related to the ***Nature of music as art***. A limited number of participants believed that due to the very specific nature of music and music-playing as an art form, certain principles from other performance domains, such as goal setting, could not directly be translated or adapted:

In a way [goal-setting] doesn’t always work for musicians, since it is an art form. I find it almost impossible to make goals measurable. And since technique and musical ideas are often so deeply connected, sometimes we don’t want to practice too specific. (TE5-*I*);

I don’t know, maybe in sports or other things, or science in general, you can break things down much easier. In music sometimes, well, if we guys tell you that we are focusing on technique or on music, you maybe take it literally actually. We are focusing on everything, but a bit more in this direction. Everything is quite unified, and you can’t just separate things. (TE3-*I*)

Finally, a ***Lack of stressful performances*** was mentioned as an intervention limitation. Some E participants argued that certain intervention principles, especially scenario planning and planned disruptions, were primarily useful for such stressful performances. However, as established professional musicians, they only rarely experienced these anymore:

I’m not planning to use the planned disruptions and what-if scenarios that quickly again. Those are things that you’ll use when you’re nervous or doing auditions. At least, that is what I think. So, I won’t use it that soon again. (E7-*I*);

I can imagine that it is more applicable when you have an important recital, audition or exam. These are key moments in which you go through one particular door, stand on one particular stage and have one particular situation. It will be more concrete in these situations. (E4-*I*)

No TE participants shared these opinions, potentially because they still experienced stressful performance moments, such as auditions. This finding might also explain why the E participants rated scenario planning significantly less favorably, compared to the TE group.

## Discussion

The aim of the present study was to conduct a process evaluation of a performance psychology intervention for both transitioning elite and elite musicians. The results show preliminary support for the perceived usefulness of the intervention. Building on previous work with music students (e.g., Clark and Williamon, 2011; Bakker et al., 2016; Hatfield, 2016), the present study highlights that performance psychology principles might not only be useful to manage performance anxiety, but can also help musicians to adopt more effective practice and performance preparation strategies. Specific positive outcomes included an increased awareness and re-examining of current practice strategies, more structured and goal-directed practice, increased practice efficiency and focus, a more proactive approach to performances, and increased attention for the physical aspects of playing. The present study also highlighted that participants demonstrated considerable variation in the methods they picked up and considered useful. This was consistent with the aim of the study, as well as that of previous interventions (e.g., Bakker et al., 2016; Spahn et al., 2016), to present participants with an amalgamation of different techniques in order to let them choose the strategies that fit best with their own goals and preferences.

### Contextual Considerations for Performance Psychology Interventions in Music

Moore et al. (2015) argued that understanding the context in which an intervention is delivered is key to understanding how and why specific interventions do or do not work in a real-world setting. From the present study, a number of contextual considerations can be derived for practitioners looking to set-up performance psychology interventions for musicians. First, in addition to the perceived outcomes, a number of additional mechanisms emerged through which performance psychology interventions might be delivered more effectively. In line with the suggestion by Clark and Williamon (2011), participants found great value in the group interactions. Interestingly, this was not only perceived as a way to learn from each other, but also to break the perceived taboo around discussing individual practice routines among high-level musicians. Somewhat in contrast to Clark and Williamon, participants also appreciated the scientific foundation of the intervention, which was believed to be largely missing in music education. Furthermore, the monitoring sessions were perceived as very valuable by the elite group, both from a motivational and a learning point of view.

Second, practitioners should carefully consider which sub-population of musicians to involve in performance psychology interventions. The majority of previous studies were targeted at music students (e.g., Clark and Williamon, 2011; Bakker et al., 2016; Hatfield, 2016). At first sight, the present findings seem to provide support for this group as primary candidate for such interventions. Scholars have found that musicians of a higher level often possess more effective practice and performance strategies (e.g., Pecen et al., 2018). As such, students would be likely to benefit more from the intervention, with some of the participants in the present study echoing this point. However, results of this study highlight there is still potential for improvement in practice and performance preparation strategies at different levels of expertise. As such, transitioning elite or elite musicians might also benefit from similar interventions, if implemented in a strategic way.

A third key contextual consideration for practitioners should, therefore, be to recognize both the abilities and the experienced challenges of their target audience. Within the present study, certain interesting differences emerged in the challenges elite and transitioning elite musicians experienced, and how certain intervention principles might be suited to address these challenges. For example, in contrast to existing research (Papageorgi et al., 2013; Kenny et al., 2014), elite musicians in our study argued that they did not experience much performance anxiety at this point in their career, and, therefore, found limited benefit in the performance preparation principles. On the other hand, the elite musicians did state that the intervention helped them to examine their old practice habits, reinvigorate their motivation, and increase their practice efficiency, even at an older age. MacRitchie and Garrido (2019) already found an inverse relationship between aging and music-specific self-efficacy in orchestra members. Thus, findings from the present study suggest that certain performance psychology principles might be suited to address such very specific challenges.

Fourth, the organizational context in which interventions are delivered should also be taken into account. Within the present study, one key difference was the way in which the intervention was set up for both groups. As highlighted in the procedure section, the intervention was offered to all transitioning elite musicians as part of the academy activities, whereas elite musicians actively enrolled. Although the intervention was voluntary for all participants, it can be expected that the difference in sampling approach led to differences in motivational profile between both groups, which in turn plays an important role in the learning process (Concina, 2019). Furthermore, the transitioning elite group experienced additional challenges related to their activities within the academy, including time constraints. Although the intervention principles were directed at increasing practice efficiency (cf. Connolly and Williamon, 2004; Hatfield, 2016), actually learning and mastering them might, paradoxically, lead to initial increased time investment. Musicians might, therefore, be expected to fall back on their old practice habits when time pressure is high and there is insufficient time to develop and gain confidence in their new skills. As such, practitioners should consider whether their intervention is provided voluntarily, how it is structurally embedded within the organization, and whether sufficient time is made available to explore, learn, and master the new practice principles to gain maximum benefits from the intervention.

Finally, cultural factors should also be considered when translating performance psychology principles to the context of music. This was exemplified by the background of the researchers being perceived as a barrier. Furthermore, a number of participants believed certain principles were difficult to apply because of the artistic nature of music making. Nevertheless, research has demonstrated the value of these performance psychology principles for musicians (Hays, 2002). As such, this point might rather reflect persisting cultural norms and beliefs, as well as a lack of adequate translation of these principles to the context of music (Pecen et al., 2016). Practitioners should, therefore, develop a clear understanding of the cultural beliefs and practices in music, and adopt the appropriate use of domain-specific terminology (Hays, 2002; Sly et al., 2019). At the same time, practitioners might also consider what Pecen et al. (2016) labeled “working *with* the culture” (p. 384) by collaborating in close relationship with the respected teachers and the existing culture; allowing the teacher to be the messenger of certain information. This also lines up with the transitioning elite musicians’ calls for the active inclusion of their mentors within the present intervention.

### Limitations and Future Directions

A number of limitations and avenues for future research should be recognized when discussing the findings of this study. First, only a limited number of participants were involved, which might limit broad statistical inferences. Although we recognize this is an important limitation, it should be noted that the statistical trends were consistent with the in-depth qualitative data gained throughout the intervention. It has been argued that such a triangulation of different data sources provides the best approach to gain an in-depth and contextualized understanding of a specific phenomenon (Creswell and Plano Clark, 2017). Nevertheless, future research would benefit from larger samples, to allow for more rigorous quantitative evaluations. Another limitation was the limited involvement of other stakeholders from the participating music organizations (e.g., management, orchestra members) in designing, implementing, and evaluating the intervention. For example, as mentor involvement was a particular theme mentioned by the transitioning elite musicians, it would have been interesting to examine the perspectives of these mentors as well. Finally, the lack of long-term follow-up of the intervention was also a limitation. Although data was collected longitudinally for a period of 10 weeks, no information was available on how participants perceived the intervention after a prolonged period of time (e.g., after 6 months) and to which extent participants are still using the included principles. As such, future intervention studies would benefit from the inclusion of such longitudinal follow-up evaluations.

## Conclusion

Performance psychology can provide an important added value for high-level classical musicians’ development and performance. Building on previous work with music students, the present process evaluation study provides preliminary support for the usefulness of a performance psychology intervention for transitioning elite and elite musicians. At the same time, a number of contextual considerations became evident when implementing this intervention within these specific populations. This information can be used by performance psychology practitioners and music educators looking to optimize future interventions for musicians of different levels of expertise.

## Data Availability Statement

The datasets generated for this study are available on request to the corresponding author.

## Ethics Statement

The studies involving human participants were reviewed and approved by Vaste Commissie Wetenschap en Ethiek van de Faculteit der Gedrags- en Bewegingswetenschappen (VCWE), Vrije Universiteit Amsterdam. The patients/participants provided their written informed consent to participate in this study. Written informed consent was obtained from the individual(s) for the publication of any potentially identifiable images or data included in this article.

## Author Contributions

JK and RO jointly designed and implemented the intervention presented in the current study. The data collection and data analysis was conducted primarily by JK, with RO acting as a critical friend and supervisor. JK produced the first draft of the manuscript, with RO providing extensive feedback.

## Conflict of Interest

The authors declare that the research was conducted in the absence of any commercial or financial relationships that could be construed as a potential conflict of interest.
